# Genetic Hitchhiking under Heterogeneous Spatial Selection Pressures

**DOI:** 10.1371/journal.pone.0061742

**Published:** 2013-04-24

**Authors:** Kristan A. Schneider, Yuseob Kim

**Affiliations:** 1 Department Fakultät Mathematik/Naturwissenschaften/Informatik, University of Applied Sciences Mittweida, Mittweida, Germany; 2 Department of Mathematics, University of Vienna, Vienna, Austria; 3 Department of Life Science and Division of EcoScience, Ewha Womans University, Seoul, Korea; UC Santa Barbara, United States of America

## Abstract

During adaptive evolutionary processes substantial heterogeneity in selective pressure might act across local habitats in sympatry. Examples are selection for drug resistance in malaria or herbicide resistance in weeds. In such setups standard population-genetic assumptions (homogeneous constant selection pressures, random mating etc.) are likely to be violated. To avoid misinferences on the strength and pattern of natural selection it is therefore necessary to adjust population-genetic theory to meet the specifics driving adaptive processes in particular organisms. We introduce a deterministic model in which selection acts heterogeneously on a population of haploid individuals across different patches over which the population randomly disperses every generation. A fixed proportion of individuals mates exclusively within patches, whereas the rest mates randomly across all patches. We study how the allele frequencies at neutral markers are affected by the spread of a beneficial mutation at a closely linked locus (genetic hitchhiking). We provide an analytical solution for the frequency change and the expected heterozygosity at the neutral locus after a single copy of a beneficial mutation became fixed. We furthermore provide approximations of these solutions which allow for more obvious interpretations. In addition, we validate the results by stochastic simulations. Our results show that the application of standard population-genetic theory is accurate as long as differences across selective environments are moderate. However, if selective differences are substantial, as for drug resistance in malaria, herbicide resistance in weeds, or insecticide resistance in agriculture, it is necessary to adapt available theory to the specifics of particular organisms.

## Introduction

When an advantageous mutation arises and rapidly increases to high frequency under strong positive selection, neutral variants on the same chromosome (initially linked to the mutation) “hitchhike” along the mutation to high frequency. This rapid change in neutral allele frequencies generates a characteristic pattern of polymorphism, commonly referred to as a “selective sweep”. Meiotic recombination breaks the association between the advantageous and the neutral allele (the “hitchhiker”). Therefore, the pattern of a selective sweep is contained within a small map distance from the locus under selection. Signatures of selective sweeps include the local reduction of polymorphism (expected heterozygosity), skew of site frequency spectrum, and a unique spatial pattern of linkage disequilibrium. As vast amounts of genome-wide data becomes available, the characteristic patterns of genetic hitchhiking provide a powerful tool to identify candidate regions in the genome that were recently (or still are) under positive directional selection. Moreover, as the quality of genetic data improves, it might be possible to develop methods aiming to reconstruct the underlying evolutionary dynamics by “reverse engineering” hitchhiking patterns. This however requires to extend classical theory to situations that reflect organism-specific characteristics regarding particularities in e.g. the selective environment, demography, or mating structure.

[1] first provided a comprehensive mathematical analysis of this evolutionary process. Since then, remarkable advancements in the mathematical theory of selective sweeps were made [2]–[5]. Theories focused on the stochastic patterns of variation, mainly achieved through coalescent and diffusion approximations, in order to detect and interpret selective sweeps from DNA sequence polymorphism. Consequently, as more genomic data became available, clear cases of selective sweeps that confirm such theoretical predictions rapidly accumulated (reviewed in [6]–[8]).

Recent theoretical studies focus on the expansion of theory beyond the “standard model” of genetic hitchhiking. The standard model assumes that an advantageous mutation, arising as a single copy on a random chromosome, increases to high frequency under constant and homogeneous selective pressure in an ideal random-mating population of constant size. This model, the basic scenario of adaptive evolution that [1] considered, is simple enough to allow the application of diffusion and coalescent approximations and thus the prediction of stochastic patterns. However, a selective sweep in a real population must occur under a very complex demographic structure and under various modes of positive selection. Application of the standard model of genetic hitchhiking to the interpretation of actual data may thus lead to a serious problem. Recent studies addressed this problem by modeling selective sweeps that occur from standing genetic variation or recurrent beneficial mutations [9], [10], under arbitrary dominance of the beneficial allele [11], under selection on a quantitative trait [12], in newly derived populations [13]–[15], in geographically structured populations [16], and under the complex life cycle of malaria parasites [17], [18].

Homogeneity of selective pressure driving the beneficial mutation to a high frequency is an important assumption in the standard model of selective sweeps. Typically this is well justified even for a population that is distributed over multiple “patches” with different selective environments, if individuals move rapidly over different patches and also mate with other individuals from other patches. In that case, the population might be modeled to be panmictic under homogeneous selective pressure, which is given by the selective advantage of the beneficial allele averaged over all patches. However, as will be argued below, if mating is restricted to between individuals within the same patch, it can alter the effective rate of meiotic recombination and thus the strength of genetic hitchhiking. This may be important for many species in which mating occurs between individuals within a restricted range (under the same selective environment) but the young offspring (or seeds) are dispersed over a much wider range. Such species include plants that reproduce frequently by self-fertilization and animals that lay eggs in common breeding sites from which the young disperses into random habitats, or agents causing vector-borne parasitic diseases. Particularly plasmodium species, parasites that cause human malaria, are further important examples: male and female gametocytes produced inside a single human host enter a mosquito's gut during the blood meal and release gametocytes, which immediately fuse and undergo meiosis, producing sporozoites that are transferred to different hosts. Therefore, given that hosts constitute heterogeneous selective environments (“patches”) for parasites, an allele under selection experiences random switches of patches over malaria transmission cycles while mating always occurs between gametocytes from the same patch. This is an important consideration for malaria parasites in which strong patterns of selective sweeps due to the evolution of drug resistance were discovered [19]–[22].

This study investigates the hitchhiking effect of a mutant allele spreading over a heterogeneous environment, which is composed of patches with different selective pressures. Averaged over all patches, the mutant allele is advantageous over the wild type. This model of selection with random dispersal over patches between generations is known as the hard-selection Levene model (cf. [23], Ch. 6). [24] originally formulated this model assuming soft selection. While typically the Levene model is considered to study the maintenance of multiple alleles at the balance of selective pressures in different patches, we consider an overall advantage of the mutant allele that ensures the rapid increase of its frequency by directional selection. Our model also differs from the Levene model in which mating between individuals occur within patches.

After formulating the deterministic model and deriving the corresponding recursion equations, we study the effect of a single locus under positive directional selection on a neutral multialelic locus. We propose an analytical solution for the equilibrium frequencies and the expected heterozygosity at the neutral locus after the sweep is complete. We further derive approximations for the equilibrium heterozygosity that are easier to interpret. In particular we want to contrast within-patch mating and mating after random dispersion over the whole population. Even further, we present stochastic simulations in comparison to the analytic results of the deterministic model.

## Methods

### Overview of the Model

Assume that a haploid sexual population disperses randomly in finitely many patches 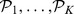. Offspring is born in a common breeding site and then migrates randomly into the 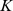 patches. Let the 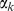 denote the proportion of individuals that migrate into patch 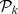. Viability selection acts differently across patches. After reaching the reproductive age adults migrate to the common breeding site for reproduction. A proportion 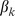 of individuals of patch 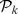 mates randomly with individuals from the same patch, whereas the remaining individuals mate randomly in the common breeding site. The haploid offspring in the next generation migrates again into the different patches from the common breeding site. The proportion 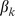 of individuals mating with other individuals of the same patch has various interpretations. It might reflect that individuals from different patches arrive at different times at the common breeding site, and hence they have a higher chance to mate with individuals from their own patch. Alternatively, it might be interpreted as matings that occur on the way to the breeding site. It might also reflect that some matings occur within the patches before migrating to the breeding site. For simplicity, we will refer to the proportion 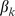 of matings, as within-patch and to the proportion 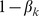 as breeding-site matings.

Suppose that the size of the population is sufficiently large to treat the evolutionary changes deterministically. Then, the population in a given generation is represented by a vector ***p*** of haplotype frequencies, which is counted after sexual reproduction in the common breeding site. The single-generation change of ***p*** is determined by the reproductive success within the different patches. Mating and meiotic recombination is as described above.

This model superficially appears to be the hard-selection Levene model (cf. [23] Chapter 6, for a diploid version), which is equivalent to the standard haploid selection model without migration. However, there is a crucial difference. Namely, the Levene model assumes that mating occurs randomly within the common breeding site, while we assume that only a proportion of individuals of each patch mates within this site. Clearly, our model reduces to the hard selection Levene model if 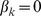 for 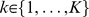. On the contrary, if 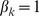 all matings occur within the patches. We will discuss the differences of our model and the hard-selection Levene model in more detail in the following sections. (The Levene model was introduced originally by [24] for soft-selection.).

### Change of Haplotype Frequencies

Assume 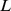 multi-allelic loci in a genome of haploid individuals, and let 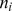 be the number of alleles segregating at locus 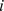, yielding to 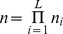 haplotypes in total. These are labelled 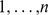 in the usual order. Their respective relative frequencies in the overall population are 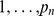, which are summarized by the haplotype-frequency vector 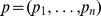.

Let 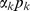 denote the frequency of haplotype 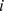 in patch 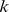. Then the (absolute) frequency of haplotype 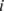 in patch 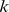 after selection is 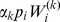. Hence, 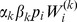, and 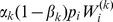 are the absolute numbers of individuals in patch 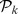 that mate randomly within the patch and at the breeding site, respectively. Moreover, 
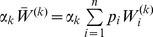
 denotes the number of haplotypes in patch 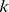 after selection. The probability that a mating between an 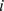
- and a 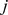-haplotype occurs in patch 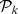 is then given by
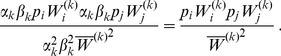
(1)


Let the probability that mating between an 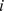
- and a 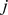-haplotype gives rise to a 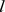-haplotype be 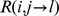. Therefore, the number of 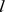-haplotypes that are produced in patch 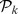 is given by
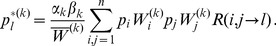
(2)


The number of 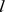-haplotypes that arrive unmated in the common breeding site is
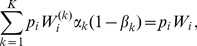
(3)where 
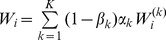
, and the total number of unmated individuals at the breeding site is



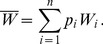
(4)Hence, the number of 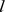-haplotypes produced in the breeding site is
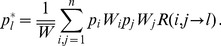
(5)


From (2) and (5), the relative frequency of haplotype 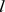 in the whole population is calculated to be
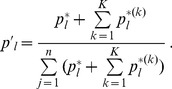
(6)


We shall briefly summarize the classical hard-selection Levene model:

#### Remark 1

*In the case of the hard selection Levene model, all individuals mate in a common pool. The relative frequency of 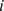-haplotypes in the mating pool is 
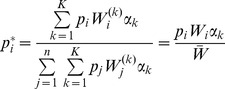
, where 
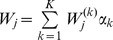
 and 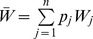. Hence, the frequency of 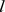-haplotypes in the next generation is given by*

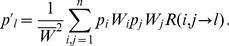
(7)


## Results

Now we want to study genetic hitchhiking, i.e., the influence of selection at a single locus on a linked neutral locus. For this purpose we assume that the first locus is selected with two alleles 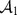 and 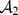, and that the second locus is selectively neutral with finitely many alleles 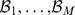. We number the haplotypes such that 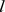 stands for 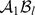 and 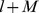 stands for 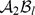 (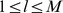). Moreover, we denote the recombination rate between the two loci by 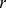.

### Dynamics at the Selected Locus

Let us denote the frequency of 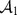 by 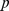 and that of 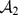 by 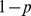. The fitnesses of a haplotype carrying the allele 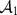 in patch 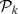 is 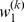, whereas that of haplotypes carrying 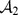 is 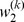. Moreover, let 
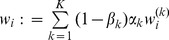
, so that we obtain 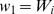 and 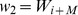 for 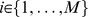.

By marginalization of the above dynamics it is straightforward to derive the dynamics for 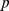. In subsection 1 of Analysis we show
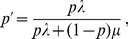
(8a)where
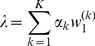
(8b)is mean fitness of 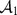 among all patches and
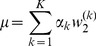
(8c)is the mean fitness of 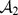 among all patches.

Note that the dynamics (8) are independent of the 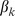
's. In particular, the dynamics (8) at the selected locus are that of the standard haploid selection model, which is identical to the hard-selection Levene model.

Summarizing, we obtain:

#### Result 1

*The allele 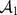 will become fixed in the population if and only if*



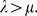
.

*Moreover, by iterating (8a) the frequency of 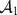 in generation 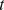, with initial condition 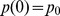, is calculated to be*

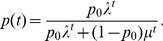
(9)


*Furthermore, we have*

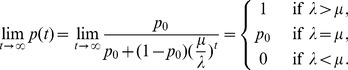
(10)


### Dynamics at the Neutral Locus

Now we want to study the hitchhiking effect of the spread of an resistant allele at a single locus on neutral variation. As before 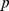 denotes the frequency of the resistant allele 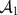. We have 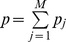. Moreover, we denote the frequencies of the neutral allele 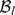 with an 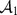-background and 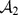-background by 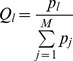, and 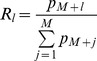, respectively.

In Analysis, subsection 2, we derive 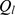 in generation 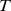 to be
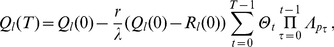
(11)where
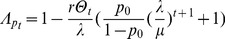
(12)and



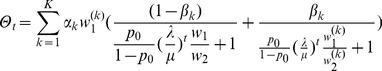
(13)

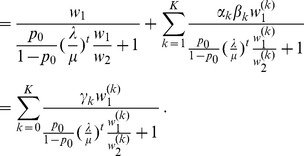
(14)


In the last step we set 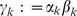 for 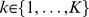, 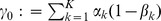, and 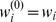. Hence, we defined patch 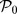 as the breeding site, and 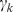 is the proportion of the population mating in path 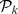.

Although, we could in principle derive 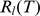 analogously, we refrain from doing so. We are only interested in the case in which the allele 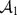 sweeps through the population. Hence, at equilibrium 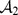 vanishes, and all neutral alleles are linked to the allele 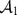. Hence, the equilibrium frequency of 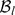 is given by 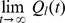. Deriving these frequencies allows to study genetic hitchhiking. In particular, we have
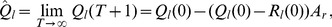
(15a)where



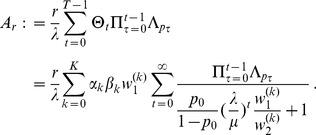
(15b)From the above it is straightforward to calculate the equilibrium heterozygosity defined by
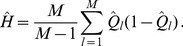
(16)


The equilibrium heterozygosity depends on the initial allele-frequency distribution at the neutral locus, because 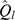 does. However, as shown in Analysis (subsection 3) the relative expected heterozygosity defined by
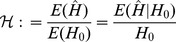



is independent of the initial distribution of allele-frequency distribution. Here, E denotes the expectation (over the initial distribution of allele-frequencies), and
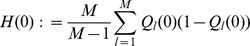
is the initial heterozygosity. We summarize

#### Result 2

*The equilibrium frequency of the neutral allele 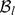 is given by (15). The expected relative heterozygosity is calculated to be*

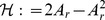
(17)
*where 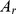 is defined in* (15b).

#### Remark 2

*For the hard-selection Levene model, we need to set* 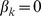 *for all* 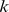 *, which gives*

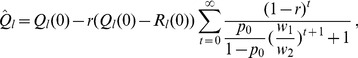
(18)
*which clearly is exactly the solution for standard hitchhiking*.

**Figure 1:**
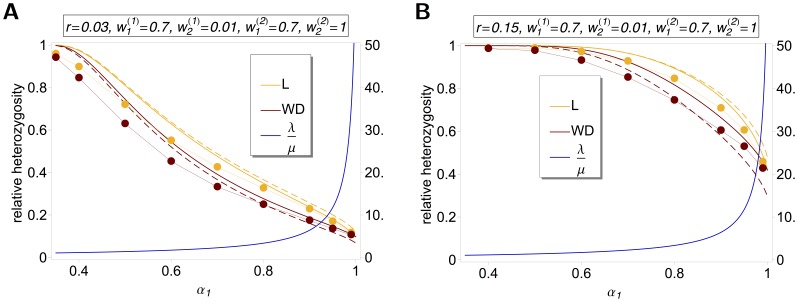
Heterozygosity as a function of 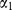. Average relative heterozygosity 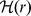 (left y-axis) and 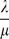 (right y-axis) as a function of 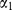 assuming two patches (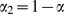). We assume either complete within-patch mating and dispersion (WD; 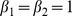) according to the model introduced here, or the hard-selection Levene model (L; 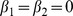). Solid lines correspond to exact solutions according to equations (15) and (18), respectively. Dashed lines show approximate solutions according to equation (25a) combined with equations (25b) and (25c), respectively. Dots represent the values obtained from stochastic simulations. Fitness values are shown in the boxes above the plot panels in (A) and (B). Stochastic simulations are based on 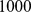 repetitions for each parameter combination and 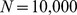. For the exact and approximate solutions we assumed 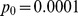 to compensate for the deterministic solution's overestimation of heterozygosity due to the prolonged initial spread of the beneficial mutation in the deterministic model.

**Figure 2:**
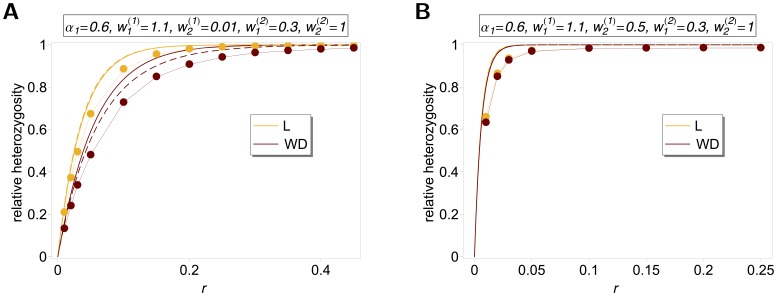
Heterozygosity as a function of 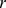. Average relative heterozygosity 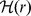 as a function of 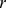. See legend of Figure 1 for more details.

**Figure 3:**
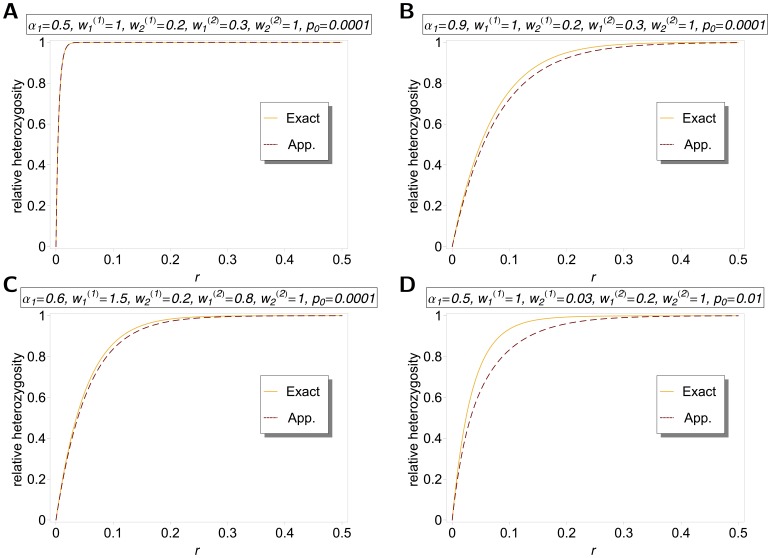
Exact *vs.* approximate average relative heterozygosity. Average relative heterozygosity 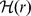 as a function of 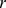 as given by (15) and (18), and equation (25a) combined with equation (25b). Two patches with 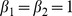 were assumed. Moreover, fitness parameters and initial frequencies are shown in the boxes above the plot panels in (A), (B), (C), and (D).

The differences between our model and the hard-selection Levene model become obvious from the above remark. Whereas the dynamics at the selected model coincide for both models, differences occur at linked neutral loci. Not surprisingly, the hard-selection Levene model is equivalent to the standard haploid selection model. In particular, the relative heterozgosity which measures the hitchhiking effect (see section 3) does not coincide for the two models. Figures 1, 2, and 3 illustrate these differences.

The analytic solution (17) is insofar not satisfying as it is iterative and difficult to interpret. We will therefore derive approximations that have a simpler form and are easier to interpret in terms of the involved parameters.

### Approximations

By writing 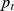 for 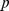, and using (8), 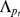 becomes
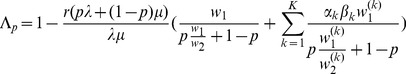
(19)(cf. 35, 28). Hence,



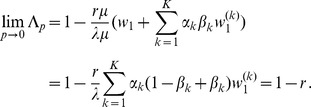
Moreover,



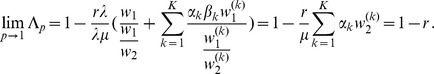



In the section Analysis we even show that 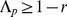, always holds. Hence, we can appoximately set 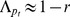. Therefore, we can approximate 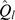 by
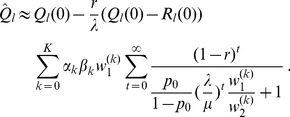
(20)


Note that (20) has the same structure as comparable quantities in [18]. Hence, (20) can be further approximated with exactly the same methods as in [18]. This leads to

#### Result 3

*The equilibrium frequency* 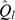 *of the allele* 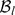 *is given by (18). If* 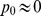 *, the frequency is approximately*

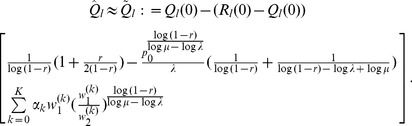
(21)


*If additionally* 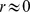 *, we approximately obtain*

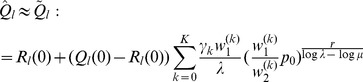
(22)

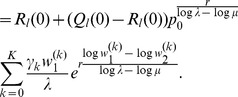
(23)


A scratch of the proof based on the results of [18] is presented in the section Analysis (subsection 4).

The above results allows for a simple interpretation. The neutral allele's frequency is a weighted average over the respective frequencies resulting from each patch (including the breeding site 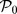). The weights are the proportion of individuals mating in each patch, 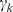, times the relative size of the patches, i.e., the relative frequency of individuals in the patch, 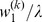. Moreover, within-patch mating leads to an adjustment factor 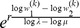 for the neutral allele's frequency within each patch as compared to standard hitchhiking. This adjustment measures how deviations of the selective regime in patch 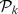 from the overall selection regime affects recombination. In particular, if 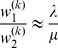, we have 
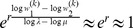
 for 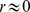. This implies that patches that reflect the population average selective pressures can be subsumed within the common breeding site. However, in patches characterized by ‘extreme’ selective regimes, deviations might be substantial. We can summarize:

#### Result 4

*Let 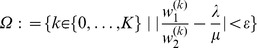 (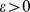), be the set of patches that reflect the overall selective regime. If 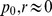, the equilibrium frequency 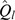 of the allele 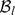 is given by*

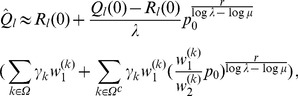
(24)
*where* 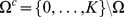.

The equilibrium heterozygosity is obtained by combining an adaptation of Result 2 with Result 3 and 4.

#### Result 5

*If* 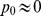 *and* 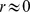 *we have*

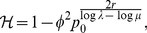
(25a)
*where*

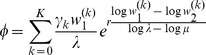
(25b)
*or*

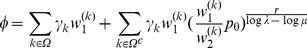
(25c)
*with* 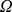 *defined as in Result 4. The factor* 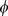 *is an adjustment due to increased inbreeding within patches caused by different survival rates resulting from different selective regimes*.

Note, that setting 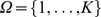 yields the approximate herterozygosity for the hard-selection Levene model. Figures 1, 2, and 3 illustrate the above result.

### Stochastic Simulations

The stochastic behavior of our two-locus model is explored by computer simulation in which the population contains a finite number (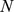) of haploid individuals. We restrict our attention to contrast the two extreme situations of complete intra-patch mating (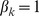 for all 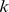) and to the hard-selection Levene model (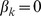 for all 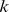). Furthermore, we will assume only two patches for most of the simulations.

Given 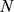 individuals in generation 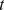, sampling of individuals (offspring) for generation 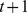 is performed in the following manner. First, a copy of a randomly-picked individual in generation 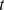 is sent to patch 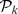 with probability 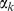. Then, a number 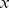 is drawn from uniform distribution between 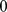 and 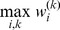. This copy is accepted (i.e. sampled) into generation 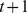 if 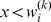, where 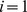 (2) if it carries the mutant (wildtype) allele. Otherwise this copy is discarded. This procedure is repeated until all 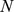 haploids are sampled. Next, to perform recombination, 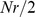 pairs of individuals are chosen and cross-overs occur. For each pair, the first individual is chosen randomly from the entire population. If 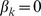, the second individual is also chosen over the entire population (Levene model). If 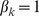, the second individual is chosen from the same patch. This completes reproduction for generation 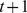. Simulations start (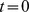) with one mutant and 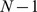 wildtype alleles. If the mutant allele is lost, the simulation starts again from the initial condition. The simulation stops when the mutant allele reaches fixation in the entire population (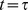). We use the method of quantifying the short-term coalescent rate from the individual-based simulation, as described in [25], to determine the expected heterozygosity at a neutral locus. Briefly, at the beginning of the simulation, all 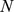 individuals carry distinct neutral alleles, as the neural allele of the 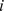
th individual is represented by the “ancestral number” 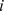 (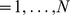). Then, let 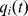 be the frequency of ancestral number 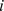 at time 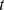 during simulation. As described above, 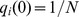 for all 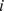. As a result of the selective sweep, 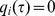 for many 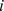, while 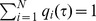. Assuming that new neutral mutations between time 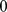 and 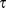 can be ignored, the expected heterozygosity at 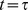 is given by
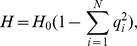
(cf. [25]).

The results of the simulation model are presented in Figures 1 and 2. As expected, the heterozygosity is lower than predicted by the deterministic model. This can be adjusted by adjusting the initial frequency in the deterministic model (i.e., by shortening the length of the trajectory).

## Discussion

While adaptive evolution in reality follows complex patterns (demography, heterogeneous selection pressures, spatial structure, mating behavior, etc.), such processes can often be accurately described within the idealized framework formed by standard population-genetic assumptions (constant homogeneous selection pressures, constant population size, random mating). Deviations from standard assumptions - particularly heterogeneities in selective pressures - are obviously important in allopatry and parapatry. However, even individuals living in sympatry might experience substantial differences in selective pressures. Examples include selection for herbicide resistance in weeds [26]–[28], stress tolerance in insects and weeds in agriculture, insecticide resistance in bed bugs [29]–[32], drug resistance in vector borne diseases (see below). Whereas in these examples candidate regions under selection might be inferred with population-genetic methods that build up on standard theory, substantial errors could result when attempting to reconstruct the underlying evolutionary dynamics (e.g., estimating selection coefficients, speed of evolution, recombination rates, etc.) from the selective sweep patterns. To avoid misinferences under such scenarios, it is therefore necessary to validate the applicability of standard population-genetic theory, and - if appropriate - adapt existing theory, particularly since many of the mentioned examples are matters of economic relevance and/or global health interest.

For instance, Plasmodium parasites causing human malaria typically experience different ‘environmental conditions’ depending on characteristics of human hosts determining selective regimes (drug treatment, drug dosage, immune response, levels of host-acquired or natural immunity, etc.). Parasites conferring resistance to antimalarial drugs are advantageous only in hosts treated with the respective drugs, whereas they are slightly deleterious in untreated hosts due to metabolic costs. In parallel, sexual reproduction occurs inside the mosquito vector, randomly but exclusively between parasites that were extracted from the same host, manifesting another deviation from standard assumption. Heterogeneous selection pressures act also on a spatial scale because drug-deployment policies and control interventions are country specific. This is particularly relevant along the borders of Cambodia, Laos, Myanmar, and Thailand where the containment of emerging artemisinin resistance is of fundamental importance to sustain successful malaria control [33]. Inferences based on standard population-genetic assumptions might be misleading as parasites experience highly varying selective environments and severe inbreeding is immanent to the specifics of malaria transmission.

More generally, parasites or pathogens that sexually reproduce within hosts might experience radically heterogeneous selection pressures, as immune responses may occur differently across organs or within specific tissues. Sexual reproduction might be common even in fungal pathogens [34]. In agriculture patches of contrasting selective regimes are created in sympatry by human interventions (cf. [35], [36]). The use of fertilizer, manure, herbicides, pesticides along with interventions such as plowing and irrigation varies across farmed land. Therefore, insects or weeds might experience radically different selective conditions across nearby acres. A striking example of a rapid evolutionary change under such a setting is the fast progression of glyphosate (“roundup”) resistance in many species of weeds, economically challenging US agriculture. Genetic understanding of glyphosate resistance will require the detection and analyses of selective sweeps in the plants, including those reproducing by self-pollination and long-distance seed dispersal.

In this study we introduced a model for heterogeneous selection in sympatry within a haploid population that randomly disperses across patches in every generation. Viability selection acts differently within the patches and mating occurs randomly within or between patches. In the limiting case that mating occurs randomly between all patches, the model reduced to the hard-selection Levene model (cf. [23], Ch. 6), which is identical to the standard selection model. However, if mating occurs exclusively within demes, the deviations from the standard model can be substantial. We showed that the dynamics at a single selected locus are independent of the dispersal pattern. Namely, they are solely determined by the average selection intensities across patches. However, as soon as two or more linked loci are considered deviations from standard-population-genetic assumptions become apparent. Particularly, we studied how the genetic variation at a neutral locus is affected as a beneficial mutation sweeps at a nearby linked locus.

We were able to derive an analytic solution for the allele frequencies at a neutral locus after the beneficial mutation became fixed. As the analytic solution is complicated we also derived approximations, which allow for clear and simple interpretations. Namely they reflect the frequency change driven by the selective pressure averaged over patches, however adjusted by a factor determining the relative importance of the patches. As long as differences in selection pressures are moderate the hitchhiking effect is accurately described by standard population-genetic theory. However, if selection pressures are extreme as it might be the case in the above mentioned examples, heterogeneities in selection pressures in combination with intra-patch mating leads to stronger reductions in genetic variation than predicted by the standard model. The reason is as follows. Radically reversing directional selection across patches leads to mating only between individuals carrying the allele that allows survival within the respective selective environments, thus greatly increasing the effect of inbreeding. Hence, meiotic recombination is less efficient to restore genetic variation. This effect however cannot be just summarized by an adjustment of the recombination rate. In fact the unique mating scheme leads to a process for which selection and recombination cannot be decoupled.

We also performed stochastic simulations to verify the results of the deterministic model's analytic prediction. As expected the deterministic solutions were underestimating the reduction of genetic variation at neutral loci. However, as usual this can be compensated by adjusting the effective initial frequency of the advantageous allele, which reflects the shorter allele frequency trajectory of the advantageous allele conditional on its escape from extinction by random genetic drift.

In general our results are informative to properly interpret selection coefficients when these are attempted to be measures from the patterns of selective sweeps. Unfortunately, appropriate data is unavailable for the mentioned examples to which our model would apply (pesticide and herbicide resistance). Nevertheless, as the examples are of great economic interest, and as population genetic theory continues to advance such data hopefully become available soon. Anyhow, the model is applicable to malaria where attempts have been made to link estimates of selection to the hitchhiking pattering (e.g. [19], [22]).

The hitchhiking effect revealed in this study might be compared to that of another study assuming the subdivision of population into many small demes or patches [16], [37]. They predicted the reduced strength of hitchhiking (higher heterozygosity), in contrast to our current result, due to population subdivision. Their model however assumes homogeneous selective pressure over demes and limited migration of individuals between demes. In such a case, the delay in the propagation of advantageous allele into the entire population provides more opportunities for recombination that breaks the hitchhiking. Most populations in nature would violate the assumptions of both studies (instantaneous dispersal among demes of the current study and homogeneous selective pressure in [16]). Further investigation is needed for the joint effect of the two forces.

## Analysis

### 1 Single-locus Dynamics

Here, we derive the marginal dynamics at a single locus. Let 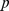 denote the frequency of allele 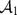, i.e., 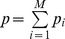. The fitness of allele 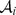 in patch 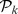 is denoted by 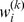. Hence, we have 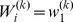 and 
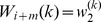
 for 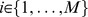. Moreover, let 
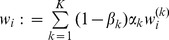
, so that we obtain 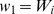 and 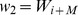 for 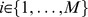.

With the above notation we can derive 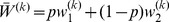. Thus,
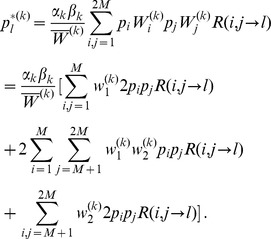





Assume 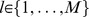. Then, by denoting the the Kronecker-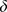 by 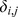 we obtain
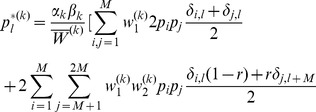


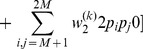


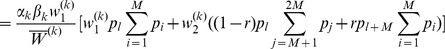


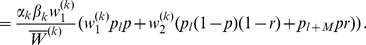



Similarly, for 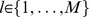, we obtain
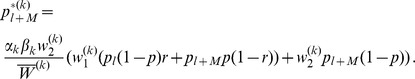



Therefore,
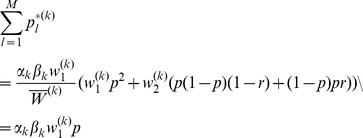
and



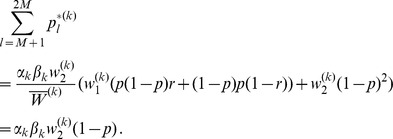



Using 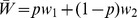 a similar calculation as above gives,
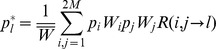


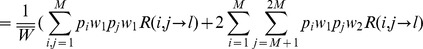


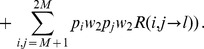



Hence, for 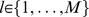, we have
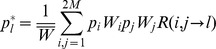


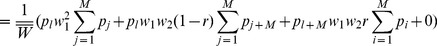


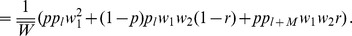



Similarly, for 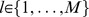, we have
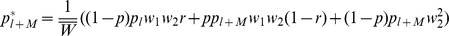



Hence,
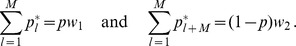



Therefore, (6) simplifies to
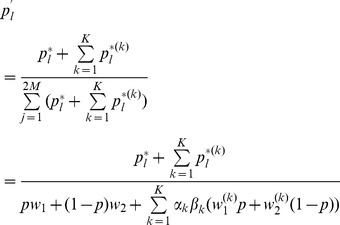


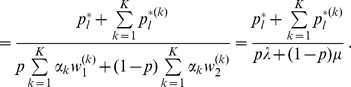



Hence, it is easily seen that
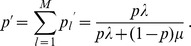



### 2 Two-locus Dynamics

Here we derive 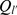 and 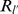. First, we need to drive 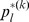 and 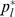 from (2) and (5), respectively. For 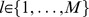, straightforward calculation (similar as in Analysis, subsection 1) yields.
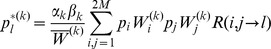


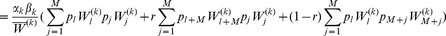


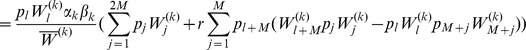


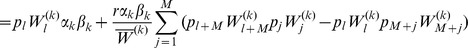


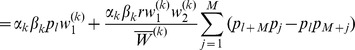


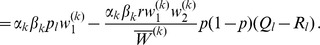



Clearly, we have
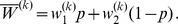



For 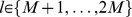 the calculation is similar. Summarizing we obtain
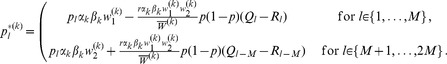



Exactly the same calculation as above yields
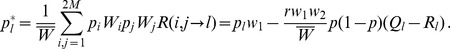



Hence,
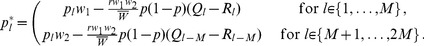
(26)


Therefore, for 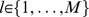, we have
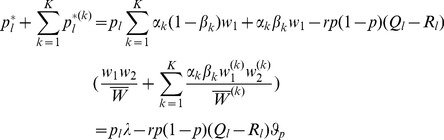
(27)where



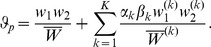
(28)Similarly, for 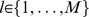, we have
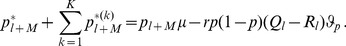
(29)


Consequently, because 
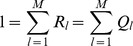
, we have
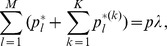


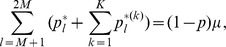


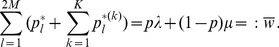
(30)


In particular, by combining (6) with (29) or (27), and (30) we obtain.
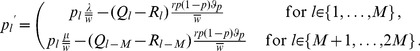
(31)


Therefore, we deduce from (26) and (31).
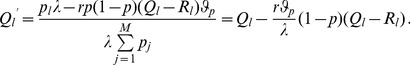
(32)


Similarly, we obtain
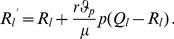
(33)


We have
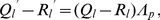
(34)where



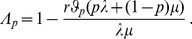
(35)Iteration of (34) yields.
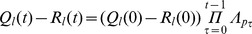
(36)


Hence, from iterating (32) using first (36) and then (28) and (9) we obtain.
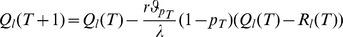


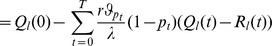


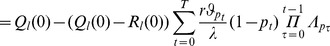


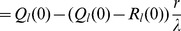


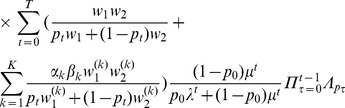





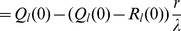
.
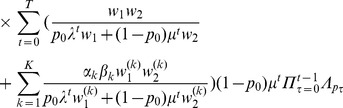


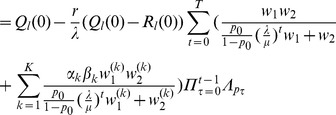


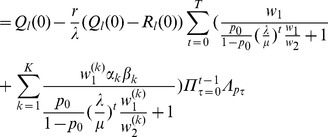


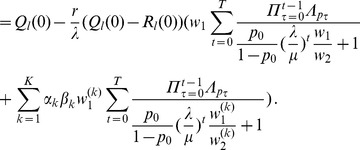



By combining (35), (28), and (9) we see that 
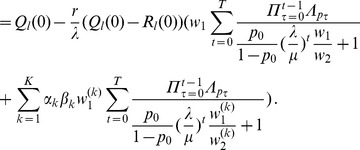
 is given by (12). Hence, the above yields (11), by substitution 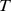 by 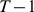.

### 3 Equilibrium Heterozygosity

Obviously, (15) has the form.
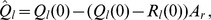
where 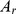 does not depend on 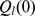 or 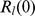. The equilibrium heterozygosity is given by



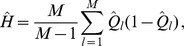
because the beneficial allele becomes fixed at equilibrium.

Now, assume that initially only a single copy of the beneficial mutation arises. Hence, we have 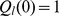 with probability 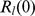 and 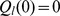 with probability 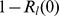, i.e., 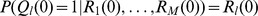 and 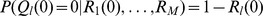. Therefore, we have.
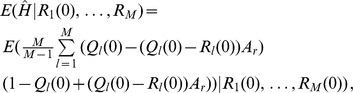


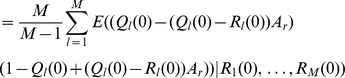


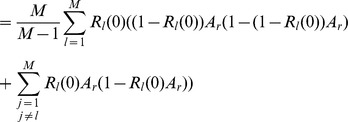


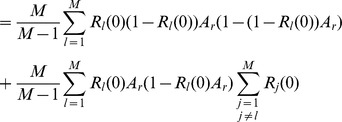


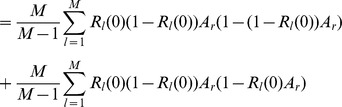


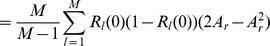


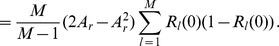



Since 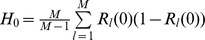, we have
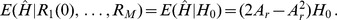



Hence we have
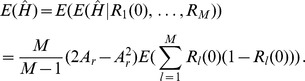



Since
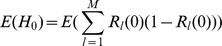
is the heterozygosity before the sweep, we see that the relative heterozygosity



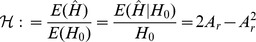
is independent of the initial allele-frequency distribution before the sweep

### 4 Approximations

Let 
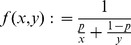
 for 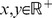. Its Hessian matrix is calculated to be
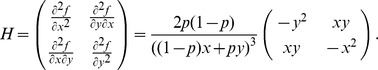



Clearly, we have 
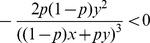
 and 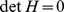, i.e., the leading minors of 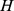 are non-positive. Hence, 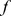 is concave but not strictly concave (note that 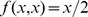). Hence, for positive random variables 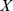 and 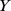 the Jensen's inequality for higher dimensions yields.
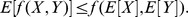



Now, choose 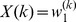 and 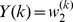 with probability 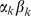 for 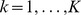, 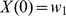 and 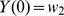 with probability 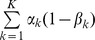. Then the Jensen's, inequality gives.
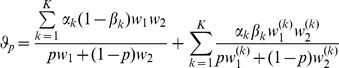


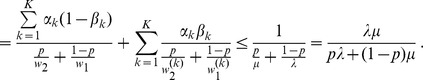



Using this inequality yields
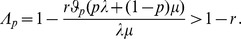



#### Proof of Result 3

First, we approximate 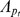 by 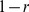. Therefore, we obtain the approximation (20), which we can rewrite as.
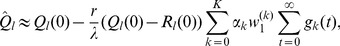
(37)with
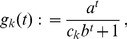
(38)where



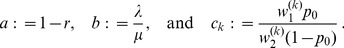
(39)As in eq. 45 in [18], we can approximate.
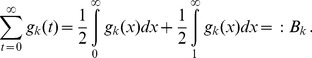
(40)


The integrals can be expressed in terms of the hypergeometric function. Exactly the same derivations as in the proofs of Theorem 1 and, Remarks 1 and 2 in [18] yield the desired expressions. The hypergeometric function can be further approximated as in the proofs of Theorem 2 and Remark 3 in [18]. Finally, assuming 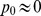, the same approximations as in Theorems 3 and 4 in [18] can be applied. According to eq. 95 in [18] we obtain.
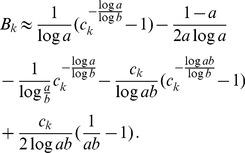
(41)


If 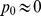, we have 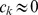. We have to keep in mind that the expressions 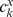 are delicate for 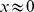. However, since 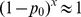, we have 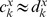, with 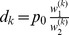. We obtain.
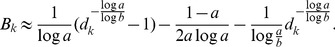
(42)


By combining (37), (40), and (43) and a little rearrangement, we obtain (21).

Furthermore, for small 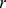 we additionally have 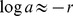, such that.
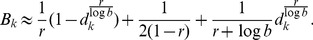
(43)


Clearly, for 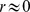, the first second and third term in the above expression are negligible compared with the first term since 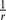 is large. Hence, we have
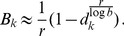
(44)


Now, (22) follows from combination of (37), (40), and (44).
